# Effect of an enhanced recovery after surgery-based multidisciplinary perioperative care pathway in children undergoing laparoscopic high ligation of the hernia sac

**DOI:** 10.3389/fsurg.2026.1940313

**Published:** 2026-09-25

**Authors:** Jia Zhou, Yanling Wu, Dongwei Lu, Lichun Dai, Xiaomeng Zhou, Lingyan Yang

**Affiliations:** 1 Department of Pediatric Surgery-Gynecology (Ward II), Affiliated Hospital of Nantong University, Nantong, Jiangsu, China; 2 Department of Gynecology (Ward I), Affiliated Hospital of Nantong University, Nantong, Jiangsu, China; 3 Department of Pediatric Surgery, Affiliated Hospital of Nantong University, Nantong, Jiangsu, China

**Keywords:** complications, enhanced recovery after surgery, laparoscopic high ligation of pediatric hernia sac surgery, pediatric inguinal hernia, perioperative nursing

## Abstract

**Aim:**

To evaluate an enhanced recovery after surgery (ERAS)-based perioperative care pathway in children undergoing laparoscopic high ligation of the hernia sac.

**Methods:**

This prospective randomized controlled trial enrolled 120 children with inguinal hernia who underwent laparoscopic high ligation of the hernia sac between March 2023 and April 2024. Participants were randomly assigned to routine perioperative care (control group, *n* = 60) or routine care plus an ERAS-based multidisciplinary perioperative pathway (study group, *n* = 60). The primary outcome was postoperative hospital stay. Secondary outcomes were recovery of bowel sounds, time to first passage of flatus, time to first oral intake, in-hospital complications, and parental satisfaction. Pain, restlessness, PSQI, and Barthel Index measures were analyzed separately as purely exploratory outcomes.

**Results:**

Mean postoperative hospital stay was 4.26 days in the study group and 6.37 days in the control group [mean difference, −2.11 days; 95% confidence interval (CI), −2.53–−1.69; Cohen's d = −1.82; *P* < 0.001]. Bowel-sound recovery, first passage of flatus, and first oral intake also occurred earlier in the study group, with mean differences of −1.42 h (95% CI, −1.99–−0.85; d = −0.89), −2.03 h (95% CI, −2.84–−1.22; d = −0.90), and −0.88 days (95% CI, −1.04–−0.72; d = −2.02), respectively (all *P* < 0.001). The in-hospital complication rate was 1.67% versus 13.33%, corresponding to an absolute risk reduction of 11.67 percentage points (95% CI, 2.03–22.59), a relative risk of 0.13 (95% CI, 0.02–0.97), and a number needed to treat of 9. Parental satisfaction was 96.67% versus 83.33% (risk difference, 13.33 percentage points; 95% CI, 2.44–24.95; *P* = 0.015).

**Conclusion:**

The ERAS-based multidisciplinary perioperative pathway was associated with shorter hospital stay, faster gastrointestinal recovery, fewer in-hospital complications, and greater parental satisfaction after laparoscopic high ligation of the hernia sac. Findings from the pain, restlessness, PSQI, and Barthel Index measures are purely exploratory.

**Clinical Trial Registration:**

https://www.chictr.org.cn/, identifier ChiCTR2300071345.

## Introduction

Pediatric inguinal hernia most often results from persistence of the processus vaginalis and typically presents as an intermittent, reducible swelling in the groin or scrotum. Incarceration may cause pain, irreducibility, intestinal obstruction, or compromised perfusion of the herniated contents (1, 2). Because pediatric inguinal hernias do not reliably resolve spontaneously, operative repair is the standard treatment after diagnosis, with timing individualized according to age, prematurity, symptoms, and incarceration risk (2, 3). Both open and laparoscopic repair are accepted approaches. Laparoscopy permits inspection of the contralateral internal ring and minimally invasive high ligation, although the choice of technique depends on the child's clinical characteristics, surgeon expertise, and institutional practice (2, 4). Regardless of approach, perioperative management should include age-appropriate assessment, analgesia, physiological monitoring, and family-centered support (5).

Enhanced recovery after surgery (ERAS) is a multidisciplinary, evidence-based approach that integrates preoperative, intraoperative, and postoperative measures to reduce surgical stress, support physiological recovery, and shorten hospitalization (6–8). In pediatric surgery, ERAS pathways may include age-appropriate fasting, active warming, multimodal analgesia, early oral intake, optimized fluid therapy, psychological support, and early mobilization (8, 9). However, evidence regarding the effectiveness of an ERAS-based perioperative pathway in children undergoing laparoscopic high ligation of the hernia sac remains limited.

This study therefore evaluated the effects of an ERAS-based multidisciplinary perioperative care pathway in this population.

## Methods

### Patients

This prospective randomized controlled trial enrolled 120 children with inguinal hernia who underwent laparoscopic high ligation of the hernia sac at our hospital between March 2023 and April 2024. Participants were assigned in a 1:1 ratio to the control group (*n* = 60) or study group (*n* = 60) using a random-number table. The allocation sequence was generated by a researcher who was not involved in recruitment or intervention delivery. Allocation was concealed in sequentially numbered, opaque, sealed envelopes that were opened only after enrollment. Participants and parents were not blinded. Surgeons, anesthesiologists, and nurses delivering or documenting the assigned perioperative care were also not blinded because the pathway required protocol-specific clinical actions. Outcome classification and data extraction were performed by trained research nurses who did not participate in intervention delivery and were blinded to group allocation; the case-report forms used coded study identifiers and did not display the assigned care pathway. After data cleaning, an independent statistician received a de-identified dataset in which the groups were labeled “A” and “B” and remained blinded to group identity until the prespecified analyses were completed. Thus, participants, parents, and the clinical care team were not blinded, whereas the independent outcome assessors and data analyst were blinded. No participant was lost after randomization, and all 120 randomized children were included in the final analysis. Participant flow and the blinding status of all parties involved in data collection and analysis are shown in Supplementary Figure 1.

Inclusion criteria were: (1) inguinal hernia diagnosed according to Practical Pediatric Surgery and confirmed by preoperative color Doppler ultrasonography; (2) age 1–12 years; (3) eligibility for elective laparoscopic high ligation of the hernia sac; and (4) unilateral disease.

Exclusion criteria were: (1) an immune or immunodeficiency disorder; (2) severe cardiac, hepatic, or other major systemic disease; (3) a communication impairment or diagnosed mental disorder that precluded study assessments; and (4) a coagulation disorder.

The study was approved by the Ethics Committee of the Affiliated Hospital of Nantong University (Approval No. 20230318-21; March 12, 2023). Written informed consent was obtained from the parents or legal guardians of all participating children.

### Surgical procedure

All children underwent laparoscopic high ligation of the hernia sac under general anesthesia with endotracheal intubation, and all procedures were performed by the same surgical team. (1) A 5-mm curvilinear incision was made at the umbilicus, and an insufflation needle was inserted to establish a carbon dioxide pneumoperitoneum. (2) A 5-mm trocar and laparoscope were introduced to identify the hernia sac and internal inguinal ring and to inspect for an occult contralateral hernia. (3) At the surface projection of the internal ring, a needle was advanced vertically through the abdominal wall to the extraperitoneal plane and then withdrawn. (4) A hernia needle loaded with 10-mm Myosel suture was passed circumferentially around the internal ring while avoiding the vas deferens and spermatic vessels. The suture was exteriorized and ligated. The incision was then closed and covered with a sterile dressing.

### Perioperative care pathways

Both perioperative pathways were implemented with support from the same nursing team, comprising one head nurse and six registered nurses, each with more than 3 years of clinical experience.

The control group received routine perioperative care.

1. Preoperative care. Nurses coordinated the required preoperative examinations, provided parents with concise information about the procedure and perioperative care, and offered age-appropriate psychological support to reduce anxiety in the child and family.
2. Intraoperative care. Before surgery, nurses prepared the operating room at 23–26 °C and approximately 50% relative humidity. During surgery, they assisted the surgical and anesthesia teams, monitored the prescribed infusion rate and volume and the child's vital signs, supported safe positioning, and documented intraoperative observations.
3. Postoperative care. Nurses assessed the incision and surrounding skin for changes in color, temperature, and swelling; administered prescribed antimicrobial or analgesic treatment when clinically indicated; monitored the child's postoperative condition; and documented or escalated suspected complications for medical review.

The study group received routine care plus an ERAS-based perioperative pathway. The principal differences from routine care were shorter preoperative fasting and fluid-restriction periods, preoperative oral glucose, active temperature management, short-acting analgesia, earlier postoperative oral intake, restrictive postoperative intravenous fluid therapy, and early mobilization. Psychological support and parental education were provided in both groups but were delivered more systematically in the ERAS group.

The ERAS protocol was a multidisciplinary perioperative pathway rather than a nursing-only intervention. Decisions regarding fasting, oral glucose administration, analgesia, and fluid management were implemented collaboratively by the nursing, surgical, and anesthesiology teams in accordance with institutional protocols. The observed effects therefore reflect the integrated pathway and cannot be attributed exclusively to nursing actions. Component-level timing, intervention specifications, progression criteria, and responsible team members are summarized in Supplementary Table 1.

1. Preoperative care. Nurses provided parents with structured education about inguinal hernia, the planned procedure, perioperative cooperation, and postoperative precautions. The operative steps, expected recovery, and practical aspects of care were explained in age-appropriate language. Parents were encouraged to use familiar, effective strategies to comfort the child and to provide reassurance and companionship. If a child had not defecated on the night before surgery, a glycerin suppository was administered when clinically appropriate. Solid food was withheld for 6 h and oral fluids for 4 h before surgery. When not contraindicated and approved by the anesthesia and surgical teams, 20–40 mL of glucose water was administered orally 4 h before surgery to reduce hunger and the risk of hypoglycemia.
2. Intraoperative care. Before surgery, skin antisepsis was performed with an appropriately warmed solution, and infused fluids were warmed with a temperature-controlled device. Nurses used calm verbal and nonverbal communication to reduce the child's anxiety during anesthetic preparation. Short-acting analgesics were selected and administered by the anesthesiology team; nurses assisted with monitoring and medication administration within their professional scope. During the procedure, nurses maintained aseptic technique, supplied instruments and materials promptly, and supported coordination between the surgical and anesthesia teams.
3. Postoperative care. (a) Basic respiratory and monitoring care. Supplemental oxygen was provided for 4–6 h, with monitoring of oxygen saturation and pulse. Nurses used age-appropriate verbal reassurance, play, and comforting gestures to reduce distress. (b) Oral intake. In the absence of nausea or vomiting 3 h after surgery, the child was offered 20–30 mL of warm water. If tolerated, liquid food was introduced 4–6 h after surgery and then advanced to a semiliquid diet. Easily digested foods, including fiber-containing fruits and vegetables when age appropriate, were encouraged. Intake was advanced cautiously during the first 1–2 postoperative days according to tolerance. (c) Pain and complication management. Nurses assessed pain at scheduled intervals and administered prescribed nonsteroidal analgesics when clinically indicated. Children were assisted with effective coughing, secretion clearance, and deep-breathing exercises as appropriate. Abdominal massage was provided only when clinically appropriate and tolerated. (d) Psychological support. Communication and distraction strategies were individualized to the child's age and emotional state and included play, conversation, preferred toys, cartoons, music, lollipops, or pacifiers when age appropriate and safe. (e) Intravenous fluid management. Postoperative intravenous fluid volume and infusion rate were adjusted according to age, weight, oral intake, and clinical status. Total fluid intake was limited to less than 500 mL/day under the study pathway, and intravenous infusion was stopped on the following day unless clinical review indicated otherwise. (f) Early mobilization. Passive limb exercises and appropriate in-bed activity were initiated according to postoperative recovery. Assisted mobilization or rehabilitation exercises began 6 h after surgery when the child was hemodynamically stable, pain was adequately controlled, and participation was safe.

### Multidisciplinary team roles

The surgical team confirmed operative eligibility, performed the procedure, reviewed postoperative surgical recovery, and determined whether complications or feeding intolerance required modification of the pathway. The anesthesiology team confirmed eligibility for the fasting and preoperative carbohydrate regimen, selected and dosed intraoperative short-acting analgesia, directed anesthetic and airway management, and collaborated in perioperative fluid and temperature management. The nursing team provided education and psychological support; verified fasting and glucose-water administration; implemented warming and monitoring; administered prescribed oxygen, analgesics, and intravenous fluids; assessed nausea, vomiting, pain, respiratory status, oral-intake tolerance, and mobility; advanced feeding and mobilization when specified criteria were met; and documented protocol delivery and bedside observations. Feeding progression and mobilization required nursing assessment together with surgical clearance when clinically indicated. Analgesic selection remained the responsibility of the anesthesiology or surgical team, with nursing administration and monitoring. Component-specific responsibilities are detailed in Supplementary Table 1.

The assigned perioperative care continued until discharge.

### Outcome measures

The primary outcome was postoperative hospital stay, defined as the elapsed time in days from the end of surgery to actual hospital discharge. The trial protocol did not prespecify a uniform discharge-readiness checklist or individual criteria such as a specified oral-intake threshold, pain-score threshold, bowel-function requirement, or parental-readiness criterion. Accordingly, this endpoint represents observed discharge timing under routine clinical decision-making rather than time to a standardized medical-readiness threshold. Secondary outcomes were gastrointestinal recovery, in-hospital complications, and parental satisfaction. Pain and restlessness scores, PSQI scores, and the Barthel Index were analyzed in a separate exploratory-outcomes section and were not treated as confirmatory endpoints.

1. Perioperative outcomes included intraoperative blood loss, operation duration, and postoperative hospital stay. In the protocol and source dataset, hospital stay was recorded in days from the end of surgery to actual discharge; it was not recorded in hours.
2. Gastrointestinal recovery outcomes included time to recovery of bowel sounds, time to first passage of flatus, and time to first oral intake. Bowel-sound recovery and passage of flatus were recorded in hours after surgery.

### Exploratory outcomes

3. Wound pain was recorded at the preoperative baseline assessment on admission, before pathway initiation, and again on the day of discharge using a 0–10 visual analogue scale (VAS), with lower scores indicating less pain. Restlessness was recorded at the same timepoints using the facial-expression-based 0–10 score specified in the original data-collection protocol, with higher scores indicating greater restlessness. Pediatric self-report pain measures have age-dependent psychometric performance, with greater uncertainty in younger children (10, 11).
4. The Pittsburgh Sleep Quality Index (PSQI) was administered at the same two timepoints as the VAS and restlessness scores—preoperative baseline at admission and the day of discharge—and was used in the original protocol to record sleep-related symptoms. It comprises 7 component scores and yields a total score ranging from 0 to 21, with higher scores indicating poorer sleep. The study records did not document whether the PSQI was completed by the child or by a parent proxy, or which language/version was used. An Italian pediatric validation study included healthy children aged 3–16 years (12).
5. The Barthel Index, likewise assessed at preoperative baseline and at discharge, was used to record functional independence in basic activities of daily living, with a maximum possible score of 100 and higher scores indicating greater independence. It was not interpreted as a direct measure of quality of life. Although the Modified Barthel Index has shown reliability and construct validity in children with selected rare disorders (13), this does not establish age-specific validity for the original Barthel Index in a general surgical cohort aged 1–12 years.

### Additional secondary outcomes

6. In-hospital complications included scrotal swelling, hydrocele, and surgical-site infection. The nursing and surgical teams assessed these events by direct clinical observation from surgery until discharge; no scheduled post-discharge follow-up was conducted. Consequently, hernia recurrence—the most clinically important delayed complication of this procedure—could not be assessed and is not included in the reported complication outcome. Initial bedside observations were documented by the unblinded clinical care team, whereas outcome classification and data extraction for analysis were completed by blinded research outcome assessors using coded case-report forms.
7. Parental satisfaction with nursing care was evaluated using the Newcastle Satisfaction with Nursing Scales (NSNS), which comprises 19 items rated from 0 to 4. Scores of 0–19 indicated “dissatisfied,” 20–57 “satisfied,” and 58–76 “very satisfied.” Total satisfaction was calculated as (number very satisfied + number satisfied)/total number×100%.

### Statistical analysis

Statistical analyses were performed using GraphPad Prism 10.0. Continuous variables were assessed for normality using the Shapiro–Wilk test and for homogeneity of variance using Levene's test. Normally distributed data are presented as mean ± standard deviation. Independent-samples t tests were used for between-group comparisons of single-time-point continuous outcomes. For outcomes measured before and after the intervention, paired-samples t tests were used for within-group comparisons and independent-samples t tests for between-group comparisons at each time point. For each continuous outcome, the between-group mean difference was reported with a two-sided 95% CI. Cohen's d was calculated using the pooled standard deviation; negative values indicate lower values in the study group. Categorical variables are presented as frequencies and percentages and were compared using the chi-square test. For complications, the absolute risk reduction was defined as the control-group risk minus the study-group risk, the relative risk as study-group risk divided by control-group risk, and the number needed to treat as the reciprocal of the absolute risk reduction, rounded up to the next whole number. The 95% CI for the risk difference was calculated using the Newcombe–Wilson method, and the relative-risk CI was calculated on the log scale. No *a priori* sample-size calculation was performed; the sample comprised all eligible participants enrolled during the study period. For the primary outcome, the observed Cohen's d was 1.82 in absolute magnitude, corresponding to *post hoc* power >99.9% with 60 participants per group and two-sided *α*=0.05. A complementary sensitivity analysis indicated that 60 participants per group provided 80% power to detect Cohen's d = 0.516. All tests were two-sided, and *P* < 0.05 was considered statistically significant.

## Results

### Baseline characteristics

Baseline age and sex were reported separately by treatment group in Table 1 and were comparable between groups (all *P* > 0.05). The cohort comprised 114 boys and 6 girls.

**Table 1:** Baseline data between the two groups.

Baseline data	Control group (*n* = 60)	Study group (*n* = 60)	*χ*^2^/t value	*P* value
Gender			0.17	0.67
Male	56 (93.33)	58 (96.67)		
Female	4 (6.67)	2 (3.33)		
Age (years)	3.92 ± 1.03	4.10 ± 1.32	0.83	0.40
Hernia site			0.30	0.57
Left side	36 (60.00)	33 (55.00)		
Right side	24 (40.00)	27 (45.00)		

Data are presented as *n* (%) for categorical variables and mean ± SD for age. SD, standard deviation.

### Perioperative indicators

Postoperative hospital stay was shorter in the study group than in the control group (4.26 ± 1.02 vs. 6.37 ± 1.29 days; mean difference, −2.11 days; 95% CI, −2.53 to −1.69; Cohen's d = −1.82; *P* < 0.001; Figure 1 and Table 2). Intraoperative blood loss (mean difference, −0.58 mL; 95% CI, −1.28–0.12; d = −0.30; *P* = 0.103) and operation duration (mean difference, −1.61 min; 95% CI, −3.72–0.50; d = −0.28; *P* = 0.133) did not differ significantly between groups. The primary-outcome effect corresponded to *post hoc* power >99.9% at two-sided *α*=0.05. The sensitivity analysis showed that 60 participants per group provided 80% power to detect Cohen's d = 0.516.

**Figure 1:**
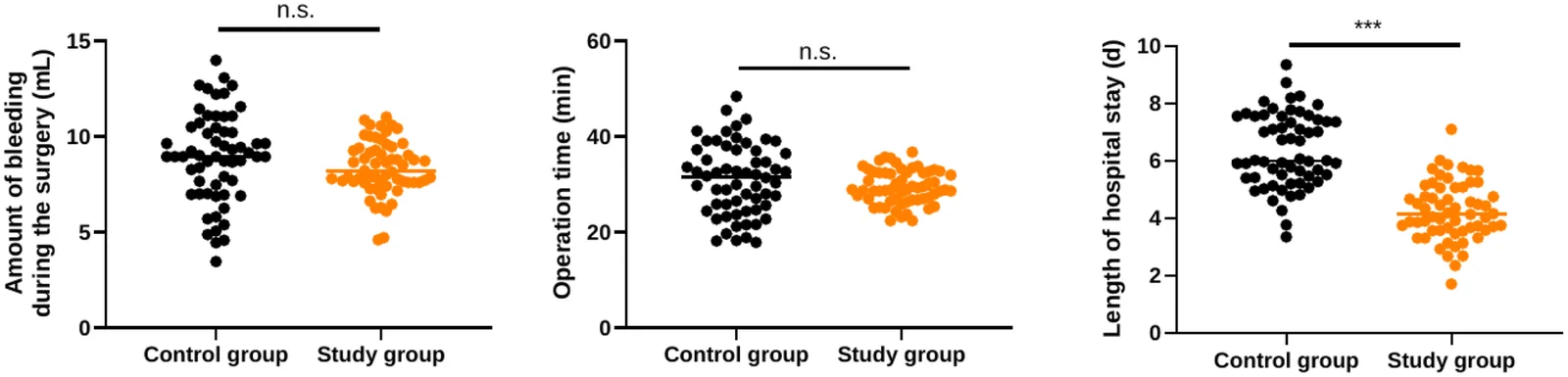
Perioperative outcomes in the two groups. Intraoperative blood loss is expressed in mL, operation duration in minutes, and postoperative hospital stay in days; the hospital-stay *y*-axis is labeled in days. Horizontal lines indicate group means. ***P < 0.001; n.s. indicates no statistically significant difference.

**Table 2:** Between-group effect estimates for perioperative and gastrointestinal recovery outcomes.

Outcome	Control group Mean ± SD	Study group Mean ± SD	Mean difference (95% CI)	Cohen's d	*P* value
Intraoperative blood loss (mL)	8.94 ± 2.35	8.36 ± 1.42	−0.58 (−1.28–0.12)	−0.30	0.103
Operation duration (min)	30.97 ± 7.44	29.37 ± 3.55	−1.61 (−3.72–0.50)	−0.28	0.133
Postoperative hospital stay (days)*	6.37 ± 1.29	4.26 ± 1.02	−2.11 (−2.53–−1.69)	−1.82	<0.001
Bowel-sound recovery (h)	8.18 ± 1.74	6.76 ± 1.43	−1.42 (−1.99–−0.85)	−0.89	<0.001
First passage of flatus (h)	12.70 ± 2.36	10.67 ± 2.14	−2.03 (−2.84–−1.22)	−0.90	<0.001
First oral intake (days)	2.75 ± 0.42	1.87 ± 0.46	−0.88 (−1.04–−0.72)	−2.02	<0.001

Data are presented as mean ± SD. Negative values indicate lower values in the study group. *Primary outcome. CI, confidence interval; SD, standard deviation.

### Gastrointestinal function indicators

Compared with the control group, the study group recovered bowel sounds earlier (6.76 ± 1.43 vs. 8.18 ± 1.74 h; mean difference, −1.42 h; 95% CI, −1.99–−0.85; d = −0.89), passed flatus earlier (10.67 ± 2.14 vs. 12.70 ± 2.36 h; mean difference, −2.03 h; 95% CI, −2.84–−1.22; d = −0.90), and commenced oral intake earlier (1.87 ± 0.46 vs. 2.75 ± 0.42 days; mean difference, −0.88 days; 95% CI, −1.04–−0.72; d = −2.02; all *P* < 0.001; Figure 2 and Table 2).

**Figure 2:**
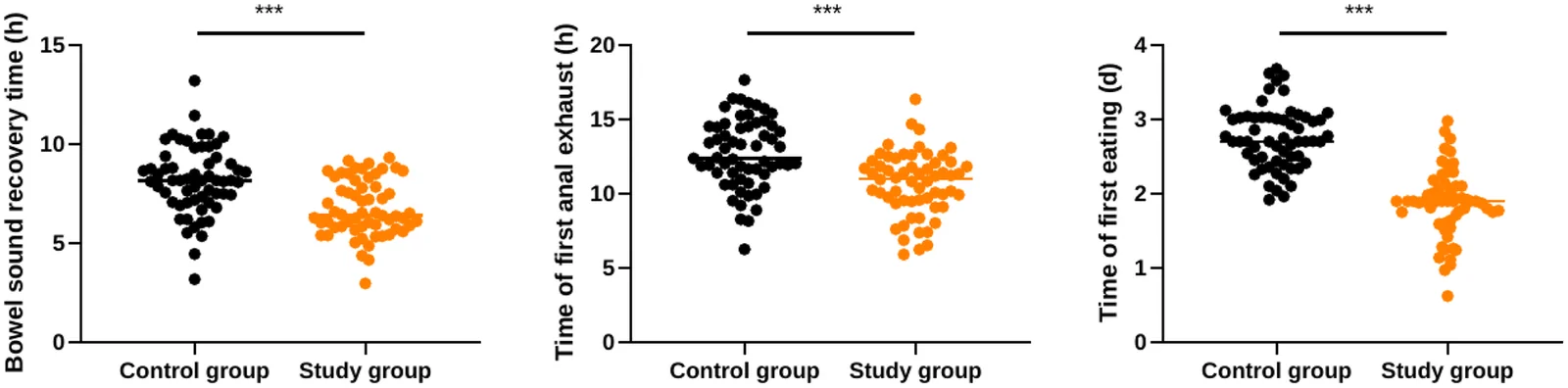
Gastrointestinal recovery outcomes in the two groups. Bowel-sound recovery and time to first passage of flatus are expressed in hours after surgery; time to first oral intake is expressed in days, and its *y*-axis is labeled in days. Horizontal lines indicate group means. ***P < 0.001.

### Exploratory outcomes

#### Pain and restlessness

Pain and restlessness scores were similar between groups at baseline (both *P* > 0.05). At discharge, pain was lower in the study group (1.11 ± 0.12 vs. 3.04 ± 0.32; mean difference, −1.93; 95% CI, −2.02–−1.84; d = −7.99), as was restlessness (2.23 ± 0.25 vs. 3.95 ± 0.42; mean difference, −1.72; 95% CI, −1.85–−1.60; d = −4.98; both *P* < 0.001; Figure 3 and Supplementary Table 2).

**Figure 3:**
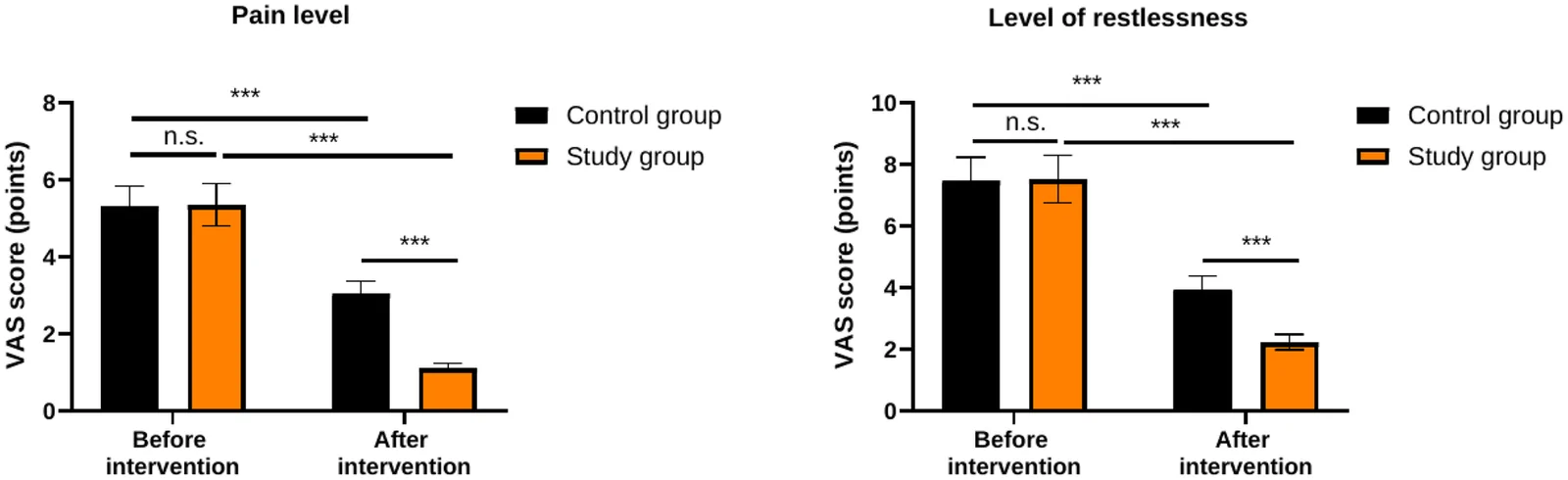
Exploratory pain and restlessness outcomes. ***P < 0.001; n.s. indicates no statistically significant difference. *P* values are nominal and unadjusted for multiplicity.

#### Sleep quality

PSQI component scores were similar between groups at baseline (all *P* > 0.05) and decreased in both groups by discharge. At discharge, mean differences (study minus control) ranged from −0.27 to −0.50 points, with all 95% CIs excluding zero and Cohen's d values ranging from −2.15 to −3.89 (all *P* < 0.001; Figure 4 and Supplementary Table 2).

**Figure 4:**
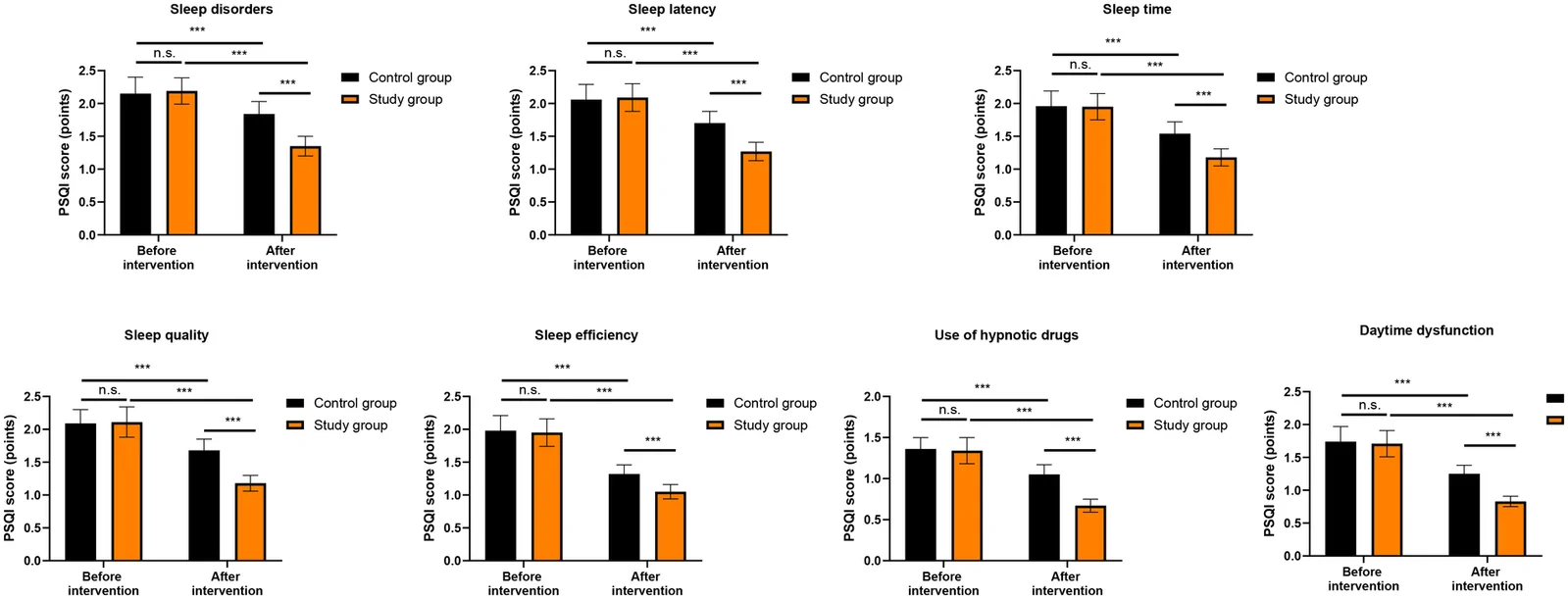
Exploratory sleep-related outcomes. ***P < 0.001; n.s. indicates no statistically significant difference. *P* values are nominal and unadjusted for multiplicity.

#### Functional independence in activities of daily living

Barthel Index scores were similar between groups at baseline (67.93 ± 6.87 vs. 68.20 ± 7.23; mean difference, −0.27; 95% CI, −2.82–2.28; d = −0.04; *P* = 0.834). At discharge, scores were higher in the study group (86.21 ± 8.65 vs. 79.92 ± 8.05; mean difference, 6.29; 95% CI, 3.27–9.31; d = 0.75; *P* < 0.001; Figure 5 and Supplementary Table 2).

**Figure 5:**
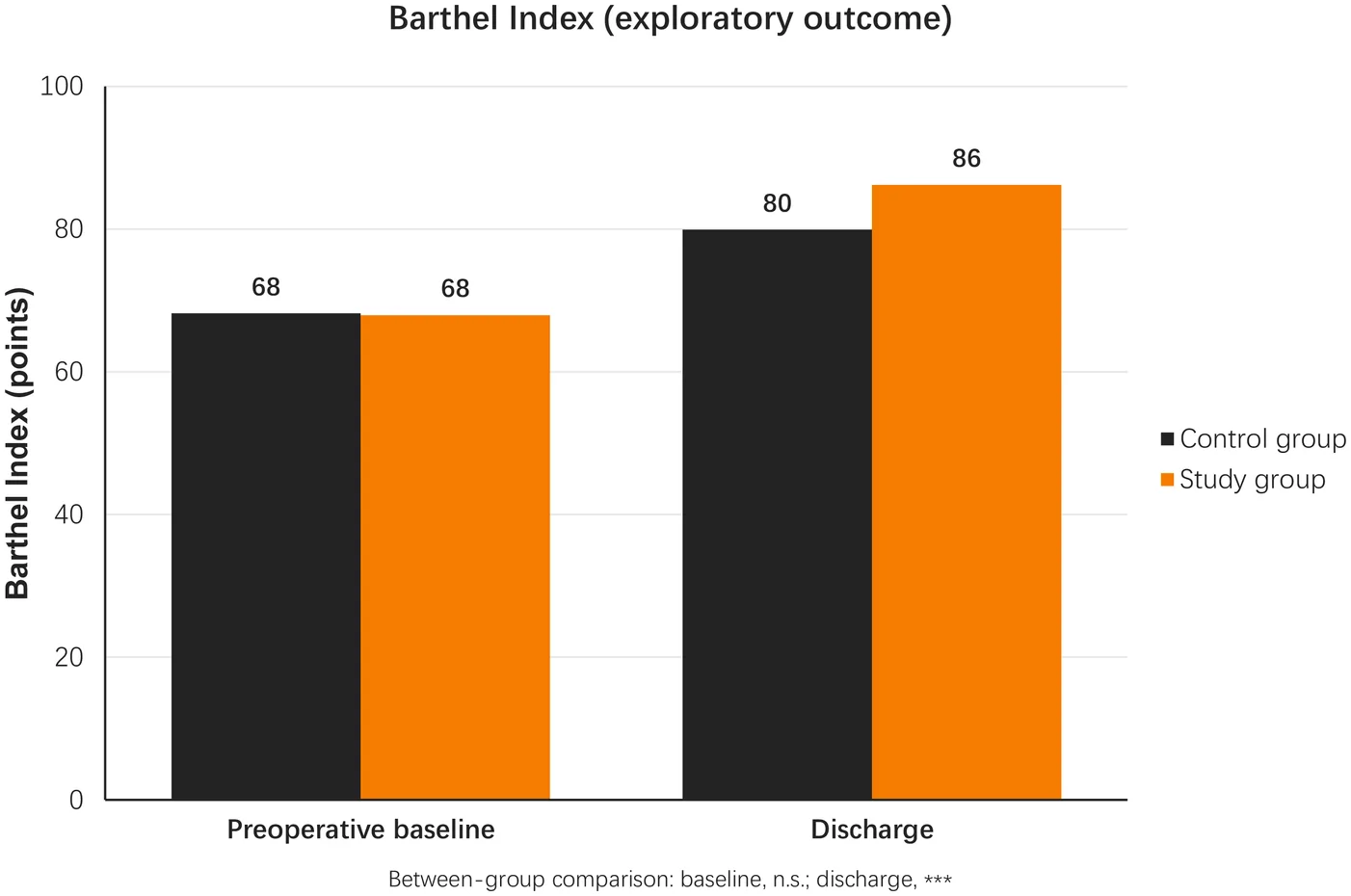
Exploratory functional-independence outcome in activities of daily living. The *y*-axis is restricted to the valid Barthel Index range of 0–100 points. Bars represent group means. ***P < 0.001; n.s. indicates no statistically significant difference.

### Additional secondary outcomes

#### Incidence of complications

In-hospital complications occurred in 1 of 60 children (1.67%) in the study group and 8 of 60 children (13.33%) in the control group. The absolute risk reduction was 11.67 percentage points (95% CI, 2.03–22.59), the relative risk was 0.13 (95% CI, 0.02–0.97), and the number needed to treat was 9 (95% CI based on the absolute risk reduction, 5–50; *P* = 0.03; Table 3).

**Table 3:** Incidence of in-hospital complications.

Groups	Cases, *n*	Scrotal swelling, *n* (%)	Hydrocele, *n* (%)	Surgical-site infection, *n* (%)	Total incidence rate, *n* (%)
Control group	60	2 (3.33)	2 (3.33)	4 (6.67)	8 (13.33)
Study group	60	0 (0.00)	0 (0.00)	1 (1.67)	1 (1.67)
χ^2^ value					4.32
*P* value					0.03

Event and satisfaction-category data are presented as *n* (%).

#### Parental satisfaction

Parental satisfaction was 96.67% in the study group and 83.33% in the control group (risk difference, 13.33 percentage points; 95% CI, 2.44–24.95; relative risk, 1.16; 95% CI, 1.03–1.31; *P* = 0.015; Table 4).

**Table 4:** Parental satisfaction with nursing care.

Groups	Cases, *n*	Very satisfied, *n* (%)	Satisfied, *n* (%)	Dissatisfied, *n* (%)	Total parental satisfaction, *n* (%)
Control group	60	23 (38.33)	27 (45.00)	10 (16.67)	50 (83.33)
Study group	60	28 (46.67)	30 (50.00)	2 (3.33)	58 (96.67)
χ^2^ value					5.92
*P* value					0.015

Event and satisfaction-category data are presented as *n* (%).

## Discussion

The ERAS concept was initially developed to improve postoperative recovery through coordinated, evidence-based perioperative management. It has subsequently been applied across thoracic, colorectal, and orthopedic surgery, with favorable effects on recovery-related outcomes (14–16). Current ERAS recommendations emphasize attenuation of the surgical stress response, maintenance of physiological function, effective analgesia, early nutrition, and timely mobilization (17). In pediatric surgery, ERAS pathways have also been associated with shorter hospitalization, earlier feeding, and improved recovery without a clear increase in adverse outcomes (18). In the present study, the principal ERAS-specific measures included shorter preoperative fasting and fluid restriction, preoperative oral glucose administration, active perioperative warming, short-acting analgesia, early postoperative oral intake, restrictive postoperative fluid therapy, and early mobilization. These measures constituted the main differences from routine perioperative care and together may have contributed to the observed postoperative benefits.

The findings are consistent with previous pediatric ERAS research. Schmoke et al. reported that an ERAS pathway in adolescents undergoing laparoscopic sleeve gastrectomy was associated with earlier oral intake, reduced opioid requirements, and shorter hospitalization (19). Shahrahmani et al. similarly found that ERAS implementation in pediatric colostomy closure reduced hospital stay, narcotic use, and time to oral feeding without increasing complications or readmissions (20). A systematic review and meta-analysis of ERAS protocols for pediatric cleft lip and palate surgery also reported shorter hospitalization, lower anesthetic exposure, reduced pain, earlier oral intake, and faster recovery (21). These studies support the broader applicability of multimodal ERAS pathways, although differences in surgical procedures, patient age, protocol composition, and discharge criteria should be considered when comparing results across settings.

ERAS programs are inherently multidisciplinary and require collaboration among surgical, anesthetic, and nursing teams. Their effects generally arise from the combined and potentially synergistic implementation of several evidence-based elements rather than from one professional discipline or a single intervention. In this study, fasting strategy, oral glucose administration, analgesic selection, and fluid management involved coordinated clinical decisions, while nurses played a central role in protocol implementation, monitoring, education, psychological support, feeding progression, and mobilization. Accordingly, the present findings support the effectiveness of the integrated ERAS-based perioperative pathway, but the independent contribution of each component cannot be determined from the current design.

No significant between-group differences were observed in intraoperative blood loss or operation duration, and both 95% CIs included zero with small standardized effects. However, postoperative hospital stay was shorter in the ERAS group by 2.11 days (95% CI, 1.69–2.53 days in absolute reduction; d = 1.82 in absolute magnitude). Postoperative hospital stay and time to first oral intake were recorded in days, not hours; thus, the reported values are not unit-labeling errors. Nevertheless, the observed stays are longer than those in many contemporary day-surgery pathways and should not be generalized to institutions using same-day discharge. This pattern suggests that the pathway primarily affected postoperative recovery and actual discharge timing rather than the technical course of the operation. The finding is consistent with a systematic review of pediatric spinal surgery, in which ERAS pathways were associated with shorter hospital stay and improved perioperative recovery (22). Because no prespecified standardized discharge criteria were documented, the estimated reduction should be interpreted as a difference in actual discharge timing that may also have been influenced by institutional practice and family readiness, rather than as a difference in a uniformly defined medical-readiness endpoint.

The ERAS group also showed earlier recovery of bowel sounds, earlier passage of flatus, and earlier oral intake. These findings may reflect the combined effects of reduced fasting, early feeding, optimized fluid administration, effective analgesia, normothermia, and early mobilization. Excessive fasting and delayed feeding can aggravate hunger, metabolic stress, and gastrointestinal dysfunction, whereas carefully monitored early oral intake may support intestinal motility and recovery. Arena et al. reported that ERAS pathways were safe and effective in pediatric gastrointestinal surgery and were associated with shorter hospitalization and faster gastrointestinal recovery (23). The present findings extend this evidence to children undergoing laparoscopic high ligation of the hernia sac, although the relative contribution of individual pathway components remains uncertain.

Pain and restlessness scores decreased in both groups and were lower in the ERAS group after intervention. This may indicate improved postoperative comfort associated with multimodal analgesia, psychological support, parental participation, and earlier restoration of normal activities. Pediatric pain assessment, however, depends on developmental stage, communication ability, and comprehension. Although ERAS-based perioperative pain management has shown favorable effects in pediatric surgical populations (24), the records did not identify the respondent or assessor for the VAS and restlessness measures and did not document the exact VAS version, age-stratified administration, or formal comprehension testing. Pediatric validation evidence is instrument- and age-specific (10, 11); therefore, these findings should be interpreted as exploratory.

The study group also had lower PSQI scores and higher Barthel Index scores after intervention. The Barthel Index reflects independence in activities of daily living rather than quality of life (25), whereas the PSQI was originally developed as a self-reported measure of sleep quality (26). Pediatric validation has been reported for the PSQI in healthy children aged 3–16 years (12) and for the Modified Barthel Index in children with selected rare disorders (13). However, these validation studies cannot establish validity for the instruments as administered in this cohort, and the findings remain supportive exploratory observations.

The in-hospital complication rate was lower and parental satisfaction was higher in the ERAS group. The complication estimate corresponded to an absolute risk reduction of 11.67 percentage points, a relative risk of 0.13, and an NNT of 9; however, the wide confidence intervals reflect the small number of events. Similar reductions in postoperative pain and complications have been reported after pediatric ERAS management for obstructive sleep apnea-hypopnea syndrome (27), and an ERAS nursing program in children undergoing surgery for supracondylar humerus fractures was associated with fewer complications and greater nursing satisfaction (28). Satisfaction could have been influenced by the inability to blind parents and care providers. These findings therefore require confirmation in larger studies.

The broad age range requires additional caution in interpretation. Children aged 1–12 years span early childhood through preadolescence and differ substantially in pain communication, perioperative anxiety, feeding behavior, mobility, and expected independence in activities of daily living. Age may therefore modify both subjective exploratory measures and clinically observed outcomes such as oral-intake progression and discharge readiness. The pooled estimates represent average effects across the enrolled cohort and should not be assumed to apply uniformly at every developmental stage. Sex-specific interpretation is also limited because only six girls were enrolled. As the trial did not prespecify subgroup hypotheses or have sufficient sample size for interaction testing, unplanned subgroup analyses would have produced imprecise estimates and an increased risk of chance findings. Future multicenter trials should stratify randomization by clinically meaningful developmental age groups and sex and prespecify adequately powered interaction analyses.

This study has several limitations. It was conducted at a single center with a modest sample size and no *a priori* sample-size calculation. Although the observed primary-outcome effect corresponded to *post hoc* power >99.9% and the sensitivity analysis indicated 80% power for d = 0.516, these analyses do not replace prospective sample-size planning and are mathematically linked to the observed effect. The confidence intervals for binary outcomes were wide because event counts were small. The pre-randomization screening log was not retained; consequently, the numbers assessed and excluded before randomization and the exclusion-reason counts are shown as NR in the CONSORT diagram and cannot be used to evaluate recruitment-stage selection. Follow-up ended at discharge, so recurrence, readmission, and other delayed outcomes were not assessed. Children aged 1–12 years were analyzed as a single cohort; no age- or sex-stratified analyses were prespecified, the study was not designed for interaction testing, and the small number of girls precluded reliable sex-specific estimates. Developmental heterogeneity is especially important for pain communication, sleep, functional independence, feeding progression, mobilization, and discharge readiness. The exploratory pain, restlessness, sleep, and functional measures also lacked documented age-specific validation and respondent/assessor procedures for the full cohort. Participants, parents, surgeons, anesthesiologists, and nurses delivering or documenting the assigned care were not blinded, although independent research outcome assessors and the statistician were blinded. Nonblinding of the clinical care team may have introduced performance bias, and parent-reported satisfaction and other subjective outcomes remained susceptible to expectation and reporting bias despite blinded outcome assessment and analysis. No prespecified standardized discharge-readiness criteria were documented, limiting reproducibility, causal interpretation, and comparison with day-surgery programs. Finally, because the ERAS pathway was delivered as a bundle, the effects of individual components could not be separated. Larger multicenter trials should use age-appropriate validated instruments with prespecified respondent/assessor procedures, prespecified and adequately powered subgroup analyses, an *a priori* sample-size calculation, prospectively standardized discharge criteria, blinded outcome assessment and analysis where feasible, and scheduled post-discharge evaluations at 6 and 12 months to assess recurrence, readmission, and other delayed outcomes.

## Conclusion

An ERAS-based multidisciplinary perioperative pathway was associated with shorter observed hospitalization, faster gastrointestinal recovery, fewer in-hospital complications, and greater parental satisfaction in children undergoing laparoscopic high ligation of the hernia sac. The pain, restlessness, PSQI, and Barthel Index findings are purely exploratory. The hospital-stay difference reflects actual discharge timing in the absence of prespecified standardized discharge-readiness criteria.

## Data Availability

The original contributions presented in the study are included in the article/Supplementary Material, further inquiries can be directed to the corresponding author.

## Supplementary material

Supplementary Figure 1 CONSORT-style participant flow diagram showing assessment for eligibility, exclusions, enrollment, allocation, follow-up through discharge, analysis, and the blinding status of participants, parents, clinical personnel, outcome assessors, and the data analyst.
