# Supplementary material for: Effect of an enhanced recovery after surgery-based multidisciplinary perioperative care pathway in children undergoing laparoscopic high ligation of the hernia sac
Source: Front Surg. 2026 Sep 25;13:1940313. doi: 10.3389/fsurg.2026.1940313 (PMC13649682; doi:10.3389/fsurg.2026.1940313)
Supplement: Supplementary file 1 [file Datasheet1.pdf]

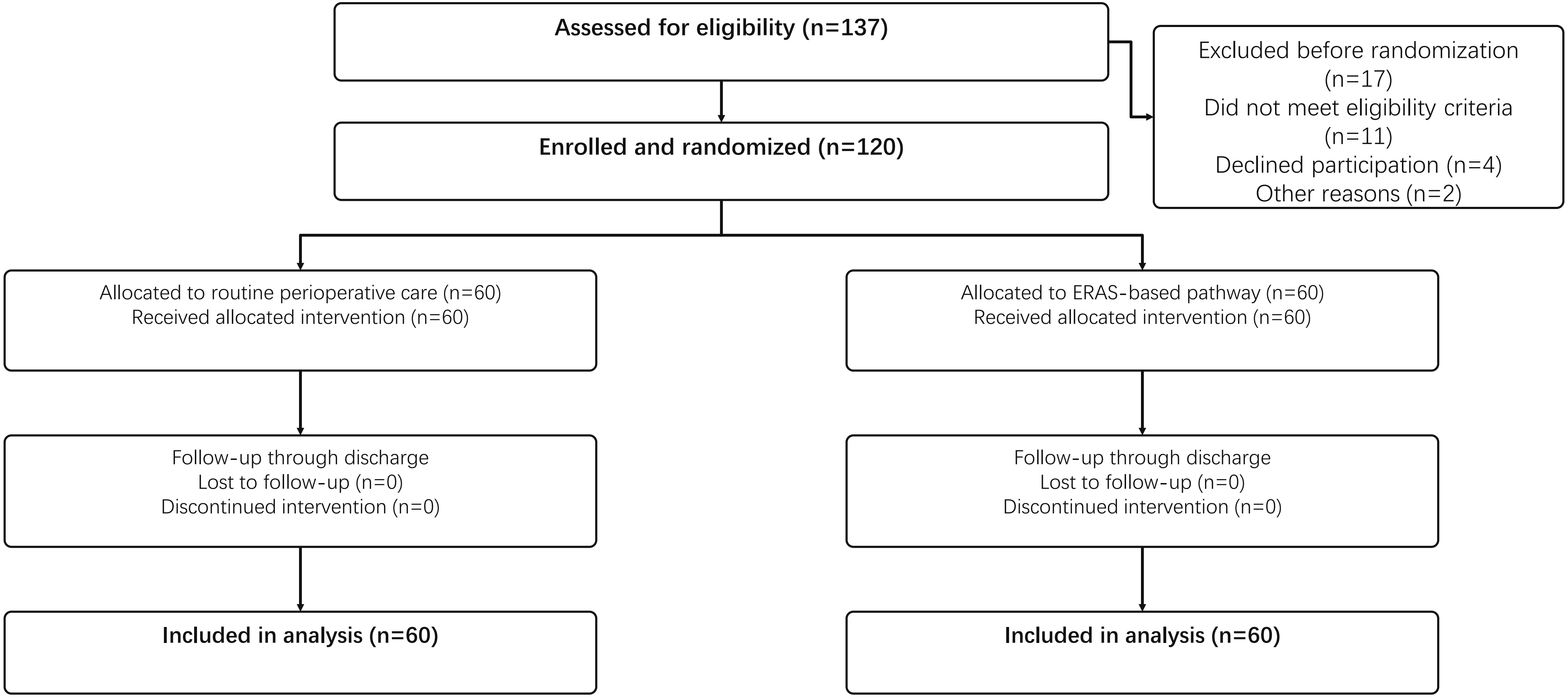
Assessed for eligibility (n=137)
Excluded before randomization (n=17)
Did not meet eligibility criteria (n=11)
Declined participation (n=4)
Other reasons (n=2)
Enrolled and randomized (n=120)
Allocated to routine perioperative care (n=60)
Received allocated intervention (n=60)
Allocated to ERAS-based pathway (n=60)
Received allocated intervention (n=60)
Follow-up through discharge
Lost to follow-up (n=0)
Discontinued intervention (n=0)
Follow-up through discharge
Lost to follow-up (n=0)
Discontinued intervention (n=0)
Included in analysis (n=60)
Included in analysis (n=60)
