# Supplementary material for: Effect of an enhanced recovery after surgery-based multidisciplinary perioperative care pathway in children undergoing laparoscopic high ligation of the hernia sac
Source: Front Surg. 2026 Sep 25;13:1940313. doi: 10.3389/fsurg.2026.1940313 (PMC13649682; doi:10.3389/fsurg.2026.1940313)
Supplement: Supplementary file 2 [file Table1.docx]

Supplementary Table 1. Structured ERAS pathway checklist and multidisciplinary responsibilities

| **Phase and component** | **Timing** | **Intervention specifications** | **Criteria for delivery or progression** | **Responsible team member(s)** |
| --- | --- | --- | --- | --- |
| Preoperative education and psychological support | During preoperative preparation | Explain disease, procedure, cooperation requirements, and precautions; guide parents in age-appropriate comforting and companionship. | Delivered after enrollment and continued according to the child’s and parents’ informational and emotional needs. | Nursing lead; surgical team available for procedure-specific clarification. |
| Selective bowel preparation | Night before surgery | Administer a glycerin suppository only when the child had not defecated. | No routine administration when the child had defecated; clinical suitability confirmed before administration. | Nursing assessment and administration; surgical team oversight. |
| Fasting and clear-fluid restriction | Before surgery | Fast from solid food for 6 h and restrict drinking for 4 h. | Schedule verified against planned anesthesia time; deviations communicated to anesthesiology and surgery. | Anesthesiology and surgery approve; nursing team instructs, verifies, and documents. |
| Preoperative oral glucose | 4 h before surgery | Administer 20–40 mL glucose water. | Proceed when oral intake is not contraindicated and the planned anesthesia schedule permits administration. | Anesthesiology/surgery confirm eligibility; nursing team administers and documents. |
| Active warming and temperature management | Before and throughout surgery | Use warmed skin disinfectant and a constant-temperature regulator for infusion; maintain operating-room temperature at 23–26°C and humidity around 50%. | Continue intraoperatively with temperature and vital-sign monitoring; adjust according to clinical status. | Anesthesiology directs physiologic management; nursing team implements warming and monitoring; surgery collaborates. |
| Short-acting analgesia | During anesthesia and as required after surgery | Use short-acting analgesia intraoperatively; assess postoperative pain and administer non-steroidal analgesics according to clinical need. | Drug selection and dose based on anesthetic/surgical assessment; nursing reassessment follows administration. | Anesthesiology leads intraoperative analgesia; surgery/anesthesiology prescribe postoperative analgesia; nursing administers and monitors. |
| Postoperative respiratory care | Immediately after surgery; oxygen for 4–6 h | Provide oxygen, monitor oxygen saturation and pulse, and guide effective coughing, sputum expectoration, and deep breathing. | Continue monitoring during the prescribed period and escalate abnormal respiratory findings. | Nursing team implements and monitors; anesthesiology/surgery review abnormalities. |
| Early oral fluids and feeding progression | 3 h and 4–6 h after surgery | If no nausea or vomiting at 3 h, give 20–30 mL warm water; at 4–6 h, progress to liquid food and then semi-liquid food. | Advance only when the preceding intake is tolerated without nausea, vomiting, abdominal distension, or other clinical concern. | Nursing team assesses tolerance and advances intake; surgical team reviews intolerance or contraindications. |
| Restrictive postoperative fluid therapy | Postoperatively; through the next day | Adjust infusion volume and rate to age and weight; keep total fluid intake <500 mL/day and stop infusion the next day. | Adjust to oral intake and clinical status; continuation beyond the next day requires clinical review. | Surgery/anesthesiology determine the fluid plan; nursing team implements, monitors, and documents intake. |
| Early mobilization | Postoperatively; assisted rehabilitation from 6 h | Begin passive limb movement and appropriate bed exercises; assist with rehabilitation exercises at 6 h. | Progress according to recovery status, hemodynamic stability, pain control, and ability to participate safely. | Nursing team leads assessment and mobilization; surgical team provides clearance when clinically indicated. |
| Complication surveillance | From surgery until discharge | Observe the incision and scrotum; monitor for scrotal swelling, hydrocele, infection, respiratory problems, pain, constipation, and flatulence. | Abnormal findings prompt surgical review and pathway adjustment. | Nursing team performs surveillance and documentation; surgical team confirms diagnosis and treatment. |

The table reports the pathway as implemented in this study. ERAS, enhanced recovery after surgery.
