# Supplementary material for: Effect of an enhanced recovery after surgery-based multidisciplinary perioperative care pathway in children undergoing laparoscopic high ligation of the hernia sac
Source: Front Surg. 2026 Sep 25;13:1940313. doi: 10.3389/fsurg.2026.1940313 (PMC13649682; doi:10.3389/fsurg.2026.1940313)
Supplement: Supplementary file 3 [file Table2.docx]

**Supplementary Table 2. Descriptive between-group effect estimates for continuous exploratory outcomes at discharge**

| **Exploratory outcome** | **Control group Mean±SD** | **Study group Mean±SD** | **Mean difference (95% CI)** | **Cohen’s d** | **P value** |
| --- | --- | --- | --- | --- | --- |
| Pain score | 3.04±0.32 | 1.11±0.12 | −1.93 (−2.02 to −1.84) | −7.99 | <0.001 |
| Restlessness score | 3.95±0.42 | 2.23±0.25 | −1.72 (−1.85 to −1.60) | −4.98 | <0.001 |
| PSQI—sleep disturbance | 1.84±0.19 | 1.35±0.15 | −0.49 (−0.55 to −0.43) | −2.86 | <0.001 |
| PSQI—sleep latency | 1.70±0.18 | 1.27±0.14 | −0.43 (−0.49 to −0.37) | −2.67 | <0.001 |
| PSQI—sleep duration | 1.54±0.18 | 1.18±0.13 | −0.36 (−0.42 to −0.30) | −2.29 | <0.001 |
| PSQI—sleep quality | 1.68±0.17 | 1.18±0.12 | −0.50 (−0.55 to −0.45) | −3.40 | <0.001 |
| PSQI—habitual sleep efficiency | 1.32±0.14 | 1.05±0.11 | −0.27 (−0.32 to −0.22) | −2.15 | <0.001 |
| PSQI—use of hypnotic drugs | 1.05±0.12 | 0.67±0.08 | −0.38 (−0.42 to −0.34) | −3.73 | <0.001 |
| PSQI—daytime dysfunction | 1.25±0.13 | 0.83±0.08 | −0.42 (−0.46 to −0.38) | −3.89 | <0.001 |
| Barthel Index | 79.92±8.05 | 86.21±8.65 | 6.29 (3.27 to 9.31) | 0.75 | <0.001 |

confidence interval; PSQI, Pittsburgh Sleep Quality Index; SD, standard deviation.
